# Recent insights into contributing factors in the pathogenesis of cirrhotic ascites

**DOI:** 10.3389/fmed.2024.1376217

**Published:** 2024-09-13

**Authors:** Zhen Li, Junfeng Zhu, Hao Ouyang

**Affiliations:** ^1^Department of Hepatology, Yueyang Hospital of Integrated Traditional Chinese and Western Medicine, Shanghai University of Traditional Chinese Medicine, Shanghai, China; ^2^Department of Hepatology, Shanghai Municipal Hospital of Traditional Chinese Medicine, Shanghai University of Traditional Chinese Medicine, Shanghai, China

**Keywords:** cirrhotic ascites, portal hypertension, renal dysfunction, inflammation, growth factors, oxidative stress, immunocytes

## Abstract

Cirrhotic ascites refers to the accumulation of fluid in the peritoneal cavity due to severe liver disease and impaired liver function, which leads to poor blood circulation in the body, increased pressure in the hepatic sinus wall, and the exudation of fluid from the plasma into the peritoneal cavity. Cirrhotic ascites is a common complication of cirrhosis and poses a threat to the health and lives of modern people, causing a heavy social burden worldwide. So far, there are no effective treatment methods available to improve the quality and quantity of life for patients and their partners; existing drugs can only alleviate the symptoms of cirrhotic ascites and slow down its progression. This article aims to carefully examine the pathogenesis of cirrhotic ascites by exploring various contributing factors such as portal hypertension, renal dysfunction, inflammation, growth factors, oxidative stress, immunocytes, and gut microbiota. The purpose is to gain better insights and deeper understanding of the mechanisms involved in this condition.

## 1 Introduction

Cirrhosis is a chronic liver disease characterized by the progressive and irreversible destruction of liver tissue, leading to the formation of scar tissue (1). It is typically caused by long-term alcohol abuse, viral hepatitis (such as hepatitis B or C), non-alcoholic fatty liver disease (NAFLD), autoimmune hepatitis, hepatocarcinoma, or genetic disorders (1). There are two main types of cirrhosis: compensated and decompensated. In compensated cirrhosis, the liver is still able to function relatively well, and patients may not show any symptoms (2). However, as the disease progresses, cirrhosis can become decompensated, leading to the development of complications such as ascites, hepatic encephalopathy, variceal bleeding, and jaundice (3, 4). The management of cirrhosis involves several strategies aimed at slowing down the progression of the disease, preventing complications, and improving the patient’s quality of life (5). One key aspect of cirrhosis management is addressing the underlying cause of the disease (6). For example, patients with alcoholic cirrhosis are advised to abstain from alcohol completely (7). Antiviral medications may be prescribed for patients with viral hepatitis to suppress viral replication (8). Another important component of cirrhosis management is the prevention and treatment of complications (9, 10).

Cirrhotic ascites is more commonly seen in patients with decompensated cirrhosis (11). This is because as the liver becomes more damaged, it is less able to effectively regulate fluid balance in the body (12). Several etiologies of cirrhosis are known to be more prone to the development of ascites. Chronic alcohol abuse is one of the leading causes of cirrhosis worldwide and is associated with a higher risk of cirrhotic ascites (13, 14). Other causes, such as viral hepatitis (particularly hepatitis C), non-alcoholic fatty liver disease (NAFLD), autoimmune hepatitis, hepatocarcinoma, and genetic disorders like hemochromatosis or Wilson’s disease, can also predispose individuals to developing ascites (14).

Cirrhotic ascites can have a debilitating impact on patients, manifesting in symptoms such as abdominal distension, eating difficulties, muscle weakness, fatigue, and respiratory distress (15). These symptoms severely limit the ability of patients to work, walk, and carry out their daily activities effectively (15). Additionally, the presence of ascites increases the risk of liver failure, infections, and other complications, further deteriorating the patient’s overall health (16). It is alarming that around 10% of cirrhosis patients develop refractory ascites, which exhibits a high incidence rate and a discouraging one-year survival rate of less than 50% (17). The consequences of cirrhotic ascites extend beyond the individual, burdening family members and caregivers who devote significant time and energy to providing care and support (18). Furthermore, it is crucial to recognize the substantial financial burden associated with treating ascites, as it places significant strain on society’s healthcare resources (19, 20).

The pathogenesis of cirrhotic ascites is multifactorial, involving complex interactions between different mechanisms. Cirrhotic ascites occurs due to a combination of factors related to portal hypertension, renal dysfunction, inflammation, growth factors, oxidative stress (OS), immunocytes, and gut microbiota. Understanding the pathogenesis of cirrhotic ascites is crucial for identifying potential biomarkers and novel therapeutic targets, advancing our knowledge and developing effective prevention strategies and therapeutic interventions to mitigate its impact on patients’ health and quality of life. Here, we summarize some of these considerable factors, emphasizing some of the recent work on cirrhotic ascites pathogenesis.

## 2 Pathogenesis of cirrhotic ascites

**FIGURE 1:**
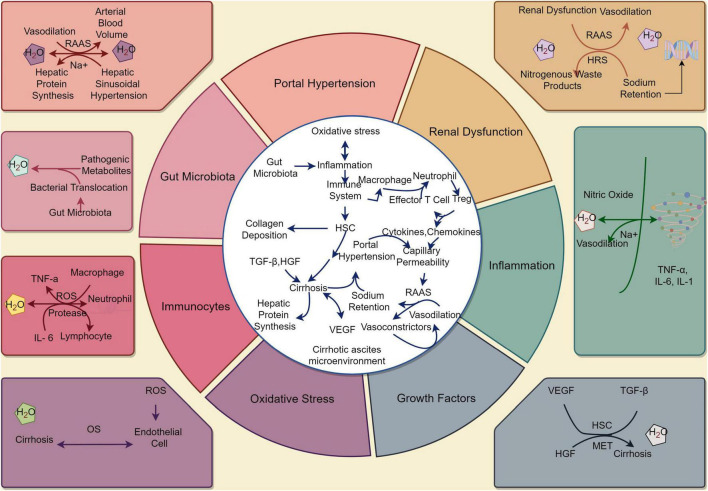
The pathophysiology of cirrhotic ascites. (By Figdraw). Under the stimulation of various factors, the stable state of the liver cell environment is disrupted, leading to the development of cirrhosis and the occurrence of ascites. These factors include portal hypertension, renal dysfunction, inflammation, growth factors, oxidative stress, immunocytes, and gut microbiota. These changes ultimately result in alterations in the normal structure and function of the liver, leading to the development of cirrhotic ascites. HSC, hepatic stellate cell; RAAS, renin - angiotensin - aldosterone system; HRS, hepatorenal syndrome; ROS, reactive oxygen species; OS, oxidative stress; TGF-β, transforming growth factor-beta; HGF, hepatocyte growth factor; VEGF, vascular endothelial growth factor; MET, epithelial transition factor; TNF-α, tumor necrosis factor-α; IL, interleukin.

Cirrhotic ascites is a multifactorial process involving the interplay of portal hypertension, renal dysfunction, inflammation, growth factors, OS, immunocytes, and gut microbiota. By understanding the underlying mechanisms and interactions between these factors, we can tailor therapeutic approaches that specifically address the complexities of cirrhotic ascites. This knowledge is essential for improving patient outcomes and reducing the complications associated with this condition. The pathogenesis of cirrhotic ascites is shown in Figure 1.

### 2.1 Portal hypertension

The portal vein is the main blood vessel that collects blood from abdominal organs, such as the intestines, spleen, and stomach, and delivers it to the liver (21). In cirrhosis, the fibrosis of liver tissue and the obstruction of blood flow lead to increased pressure in the portal vein, known as portal hypertension (22).

Portal hypertension leads to the formation of collateral vessels, which are abnormal blood vessels that bypass the liver and allow blood to flow around it. These collateral vessels divert blood away from the liver, which subsequently reduces renal perfusion (23). The decrease in renal blood flow compromises the kidneys’ ability to function properly, leading to renal dysfunction (23, 24). The renal dysfunction stimulates the renin - angiotensin - aldosterone system (RAAS) and sympathetic nervous system, leading to sodium and water retention (25–27). The increased sodium reabsorption in the proximal tubules of the kidneys results in increased intravascular volume and exacerbates the development of ascites (28). Moreover, the structural changes in the liver parenchyma, such as fibrosis and nodular regeneration, lead to increased resistance to blood flow within the liver sinusoids (29). This condition, known as hepatic sinusoidal hypertension, contributes significantly to the development of ascites (30, 31). The compromised sinusoidal integrity results in increased portal pressure, further exacerbating splanchnic vasodilation and sodium retention (32, 33). Additionally, portal hypertension also causes an elevation in the capillary filtration pressure within the splanchnic circulation (34). The increased hydrostatic pressure forces fluid through the endothelial fenestrations and into the interstitial space, resulting in the accumulation of fluid within the peritoneal cavity (35). Furthermore, the subsequent decrease in oncotic pressure due to reduced hepatic protein synthesis impairs the reabsorption of fluid from the peritoneal cavity (35).

In conclusion, there is a close relationship between portal hypertension and cirrhotic ascites. Portal hypertension is caused by the increase in pressure within the portal vein and leads to the formation of collateral circulation. This process interacts with factors such as renal dysfunction and the activation of RAAS. The treatment of cirrhotic ascites involves not only alleviating symptoms but also controlling portal hypertension and correcting liver dysfunction.

### 2.2 Renal dysfunction

Renal dysfunction or impaired kidney function can worsen the development and progression of cirrhotic ascites through various mechanisms.

One of the key reasons is the disruption in the RAAS - a hormone system responsible for regulating blood pressure and fluid balance in the body (36). In cirrhosis, the RAAS system is dysregulated, leading to increased production of vasodilators and decreased production of vasoconstrictors (37). This leads to systemic vasodilation and a decrease in effective arterial blood volume (38). The kidneys perceive this decrease in blood volume and activate compensatory mechanisms to retain sodium and water in an effort to restore blood volume (39). However, in the setting of renal dysfunction, these compensatory mechanisms can be impaired. The kidneys are unable to properly respond to the decreased blood volume, resulting in inadequate sodium and water excretion (40). This further exacerbates the fluid overload and contributes to the development of cirrhotic ascites (41). Additionally, the impaired renal function can lead to the retention of nitrogenous waste products, such as urea and creatinine (42). These waste products can accumulate in the body, leading to the development of hepatorenal syndrome (HRS) (43). HRS further worsens the fluid imbalance seen in cirrhotic ascites (44). The diminished kidney function in HRS results in the accumulation of sodium and water, leading to an elevation in blood volume and portal hypertension. Consequently, this facilitates the buildup of fluid in the abdominal cavity (26, 45).

In summary, renal dysfunction can play a significant role in inducing and worsening cirrhotic ascites. Impaired kidney function disrupts the normal regulation of fluid balance in the body, leading to fluid accumulation in the abdominal cavity. Understanding the underlying mechanisms and implementing appropriate management strategies are essential in addressing this complex condition and improving patient outcomes.

### 2.3 Inflammation

Inflammation is a fundamental immune response that occurs in the body as a protective mechanism against harmful stimuli such as pathogens, tissue damage, or irritants (46). Chronic liver injury leads to the release of pro-inflammatory cytokines and chemokines, which attract immunocytes to the liver. These immunocytes, such as macrophages and lymphocytes, further promote the formation of cirrhotic ascites by enhancing the inflammatory response (47–49).

In cirrhosis, chronic liver inflammation triggers a complex cascade of events that involve the activation of immunocytes, the release of pro-inflammatory cytokines, and the recruitment of inflammatory cells to both the liver and peritoneum (50, 51). Notably, pivotal cytokines such as tumor necrosis factor-alpha (TNF-α), interleukin-6 (IL-6), and interleukin-1 (IL-1) play crucial roles in this inflammatory response (52, 53). These cytokines play significant roles in the development of cirrhotic ascites by inducing vasodilation, enhancing capillary permeability, and activating the RAAS that promote sodium and water retention (53–57). TNF-α, in particular, stimulates the production of nitric oxide, which causes splanchnic and systemic vasodilation (58–60). This leads to further decreases in effective arterial blood volume and activates compensatory mechanisms that promote sodium and water retention (61, 62). In addition, inflammation contributes to the pathogenesis of cirrhotic ascites by promoting the formation of fibrous tissue and collagen deposition within the liver. This process, known as hepatic fibrosis, is driven by activated hepatic stellate cells (HSCs) and pro-inflammatory mediators (such as TNF-α and IL-6) (63). The progressive fibrotic changes in the liver not only lead to decreased liver function, but also induce renal dysfunction. These factors further exacerbate portal hypertension, ultimately resulting in the development of cirrhotic ascites (64, 65).

By and large, inflammation plays a critical role in the development and progression of cirrhotic ascites. The dysregulated inflammatory response in liver cirrhosis leads to the activation of immunocytes, release of pro-inflammatory mediators, and disruption of fluid homeostasis. Understanding the relationship between inflammation and cirrhotic ascites can aid in the development of targeted therapeutic strategies to mitigate its impact on patients with liver cirrhosis.

### 2.4 Growth factors

Growth factors are signaling molecules that play a crucial role in various cellular processes, including cell proliferation, differentiation, migration, and apoptosis (66). In the context of liver disease, dysregulated growth factor signaling has been shown to contribute to hepatic fibrosis, angiogenesis, and the development of portal hypertension and cirrhotic ascites (67–69).

The imbalance between pro- and anti-fibrotic growth factors has been suggested to play a critical role in the development of hepatic fibrosis, which is a hallmark of chronic liver disease. Transforming growth factor-beta (TGF-β) is one of the most potent pro-fibrogenic growth factors involved in the activation of HSCs and the promotion of fibrosis (70). It has been demonstrated that TGF-β signaling is upregulated in cirrhosis and contributes to the development of ascites through the promotion of fibrogenesis and the disruption of hepatic microcirculation (71). On the other hand, hepatocyte growth factor (HGF) is an important anti-fibrotic growth factor that acts as a potent inhibitor of HSC activation and collagen synthesis. HGF is produced by mesenchymal cells, including HSCs, and exerts its effects through the mesenchymal to epithelial transition factor (MET) receptor on hepatocytes and other cell types (72). Reduced production or impaired signaling of HGF has been observed in cirrhosis, and this dysregulation may contribute to the development of ascites by promoting fibrosis and impairing liver regeneration (73).

In addition to their role in fibrogenesis, growth factors have also been implicated in the regulation of vascular tone and angiogenesis, which are important factors in the development of portal hypertension and subsequent cirrhotic ascites formation. Vascular endothelial growth factor (VEGF) is a major angiogenic factor that stimulates the formation of new blood vessels and increases vascular permeability (74). Increased levels of VEGF have been found in the serum and ascitic fluid of patients with cirrhotic ascites, and this dysregulation may contribute to the development of cirrhotic ascites by promoting neovascularization and endothelial dysfunction (75).

In a nutshell, the dysregulation of growth factor signaling plays a crucial role in the pathogenesis of cirrhotic ascites. Imbalances between pro- and anti-fibrotic growth factors, such as TGF-β and HGF, contribute to hepatic fibrosis and impaired liver regeneration, while dysregulated angiogenic factors, such as VEGF, promote neovascularization and increased vascular permeability. Understanding the relationship between growth factors and cirrhotic ascites may provide valuable insights into the development of targeted therapeutic interventions for this condition. For example, targeted therapies that inhibit VEGF or its receptors have shown promising results in reducing ascites formation in experimental models and clinical trials (75, 76).

### 2.5 Oxidative stress

Oxidative stress has been implicated in the pathogenesis of various liver diseases, including cirrhotic ascites. The liver plays a vital role in detoxification and metabolism, which exposes it to a higher concentration of reactive oxygen species (ROS) (77). The excessive production of ROS can lead to oxidative damage to various cellular components, including lipids, proteins, and DNA (78).

In cirrhotic ascites, OS is believed to contribute to the progression of hepatic fibrosis, which eventually leads to cirrhosis (79). Studies have shown that ROS can contribute to endothelial dysfunction, inflammation, which are responsible for the activation of HSCs and the production and deposition of collagen (80–82). This activation process promotes hepatic fibrosis and contributes to the development of cirrhosis and cirrhotic ascites (83, 84). Moreover, OS can also directly affect the function and integrity of endothelial cells lining the blood vessels in the liver (79, 85). The dysfunction of these endothelial cells leads to an abnormal increase in both vasodilation and vascular permeability, which significantly contributes to the leakage of fluid into the abdominal cavity (86). This, in turn, causes an elevation in vascular resistance and ultimately leads to the development of portal hypertension (87, 88). Consequently, there is a buildup of cirrhotic ascites due to the heightened hydrostatic pressure (89, 90). Additionally, OS-induced activation of the RAAS has been shown to play a role in sodium and water retention, further contributing to the development of cirrhotic ascites (91–93).

On the whole, OS plays a significant role in the development and progression of cirrhotic ascites. Excessive generation of ROS and inadequate antioxidant defense contribute to endothelial dysfunction, HSC activation, hepatic fibrosis, and fluid retention. Targeting OS pathways may provide new therapeutic strategies for the management of cirrhotic ascites. However, more research is needed to fully understand the complex interplay between OS and cirrhotic ascites formation in liver cirrhosis.

### 2.6 Immunocyte

Immunocytes play a crucial role in the development and progression of cirrhotic ascites. In the early stages of liver cirrhosis, chronic inflammation is triggered by various factors such as alcohol abuse, viral hepatitis, or autoimmune disorders (94–96). This inflammation activates immunocytes, including macrophages, neutrophils, and lymphocytes, which are key players in the immune response (97–99).

Macrophages are immunocytes that respond to inflammation and infection. In cirrhotic ascites, activated macrophages release pro-inflammatory cytokines, such as TNF-α and IL-6, which contribute to the development of cirrhotic ascites (49). These cytokines promote the activation and migration of other immunocytes, leading to increased vascular permeability and fluid accumulation in the abdominal cavity (100–102).

Neutrophils, another type of immunocyte, are also involved in the pathogenesis of cirrhotic ascites. Inflammation stimulates the release of chemokines, which attract neutrophils to the site of injury or infection (103). Once in the liver, neutrophils promote further inflammation and OS by releasing ROS and proteases (104–106). These substances damage liver tissue and contribute to the development of ascites (91, 105, 107, 108).

Lymphocytes, particularly T cells, also play a crucial role in the development of cirrhotic ascites. The chronic inflammation in the liver promotes the activation and expansion of T cells, leading to an imbalance between regulatory T cells (Tregs) and effector T cells (109, 110). This imbalance favors the development of cirrhotic ascites by impairing the immune response and promoting the accumulation of fluid in the abdominal cavity (111).

Moreover, immunocytes are not only involved in the development but also in the resolution of cirrhotic ascites. During the resolution phase, macrophages switch from a pro-inflammatory to an anti-inflammatory phenotype (112). Anti-inflammatory cytokines, such as interleukin-10 (IL-10), are released, which reduce inflammation and promote tissue repair (49, 113). Similarly, regulatory T cells play a role in dampening the immune response, thereby reducing inflammation and promoting the resolution of cirrhotic ascites (114).

To sum up, immunocytes play a crucial role in the development and progression of cirrhotic ascites. Activation of macrophages, neutrophils, lymphocytes, and dysregulation of immune responses contribute to the development and resolution of cirrhotic ascites through the release of pro-inflammatory and anti-inflammatory molecules. Understanding the complex interactions between immunocytes and ascites formation may pave the way for new therapeutic approaches targeting the immune system for the management of cirrhotic ascites. In particularly, modulation of immunocyte function or inhibition of specific pro-inflammatory cytokines could potentially reduce the severity and frequency of ascites episodes (93, 115).

### 2.7 Gut microbiota

**FIGURE 2:**
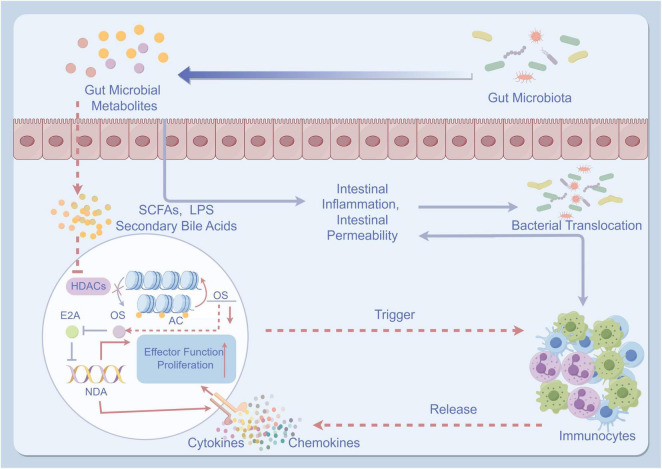
The pathogenesis of gut microbiota in the development of cirrhotic ascites. (By Figdraw). The pathogenesis of gut microbiota in the development of cirrhotic ascites involves the imbalance of intestinal microbiota, leading to impairment of the intestinal mucosal barrier and reduced intestinal barrier function. This imbalance further promotes the translocation of bacteria and toxins. These bacteria and toxins activate immune, inflammatory responses, and oxidative stress, ultimately resulting in liver dysfunction and the formation of ascites. TNF-α, tumor necrosis factor-α; IL, interleukin; HDACs, histone deacetylases; AC, adenylate cyclase; OS, oxidative stress; SCFAs, short-chain fatty acids; LPS, lipopolysaccharides.

Gut dysbiosis refers to an imbalance in the composition and function of the gut microbiota. In healthy individuals, the gut microbiota consists of a diverse community of microorganisms that play a crucial role in maintaining gut homeostasis and overall health (116, 117). However, in patients with cirrhosis, gut dysbiosis is commonly observed (118). This dysbiosis is characterized by an increase in potentially harmful bacteria, a decrease in beneficial bacteria, and alterations in microbial metabolites (119). The pathogenesis of gut microbiota in the development of cirrhotic ascites is illustrated in Figure 2.

The relationship between gut dysbiosis and cirrhotic ascites is thought to be bidirectional. On one hand, the presence of cirrhotic ascites can lead to changes in the gut microbiota composition (120). The accumulation of fluid in the peritoneal cavity can cause increased intestinal permeability, also known as “leaky gut” (121). This increased permeability allows bacteria and bacterial products to translocate from the gut lumen into the systemic circulation, leading to a systemic inflammatory response (115, 122). This translocation of bacteria and bacterial products is known as bacterial translocation and is a hallmark of advanced cirrhosis (123). Bacterial translocation can further exacerbate gut dysbiosis and contribute to the development and progression of cirrhotic ascites (41, 93).

On the other hand, gut dysbiosis can also contribute to the development of cirrhotic ascites. Dysbiotic gut microbiota produce more pathogenic metabolites such as short-chain fatty acids (SCFAs), lipopolysaccharides (LPS), and secondary bile acids compared to a healthy gut microbiota (124–126). These metabolites have been shown to induce intestinal inflammation and increase intestinal permeability, leading to bacterial translocation (127, 128). Furthermore, dysbiotic gut microbiota not only triggers inflammation throughout the body but also activates immunocytes, leading to the production of pro-inflammatory cytokines (such as TNF-α and IL-6) and chemokines (129, 130). Additionally, it induces OS, which further worsens the development of cirrhotic ascites (44, 131, 132).

Overall, gut dysbiosis plays a significant role in the development and progression of cirrhotic ascites. The bidirectional relationship between gut dysbiosis and cirrhotic ascites highlights the importance of incorporating strategies to modify the gut microbiota in the management of patients with cirrhosis. Further research is needed to fully understand the mechanisms underlying this relationship and to develop targeted therapeutic interventions for the treatment of cirrhotic ascites. For instance, probiotics, prebiotics, or fecal microbial transplantation have shown promise in improving gut barrier function and reducing inflammation in animal models of cirrhosis (118, 133). By restoring a healthy balance of gut bacteria, it may be possible to restore gut homeostasis and reduce the risk of bacterial translocation and subsequent ascites formation (44).

## 3 Conclusion and discussion

The mechanism study of cirrhotic ascites is a complex and challenging field. By studying in-depth factors such as portal hypertension, renal dysfunction, inflammation, growth factors, OS, immunocytes, and gut microbiota, as well as their interrelationships, we can better understand the pathogenesis of cirrhotic ascites and provide effective targets and strategies for its treatment.

Despite some progress in understanding the mechanisms of cirrhotic ascites, there are still many unresolved issues. Firstly, the interaction between different mechanisms and their relative importance in the development of cirrhotic ascites is still not fully clear. Cirrhotic ascites is not caused by a single factor, but rather by complex pathological and physiological changes resulting from multiple factors. Therefore, further research is needed to investigate the interactions between different mechanisms and their relative contributions in the formation of cirrhotic ascites.

Secondly, early diagnosis and prevention of cirrhotic ascites remain challenging. Currently, abdominal ultrasonography and abdominal paracentesis are commonly used for diagnosing cirrhotic ascites, but they still have limitations. Therefore, it is of great significance to identify new biomarkers and imaging techniques to improve the early diagnosis and prevention of cirrhotic ascites.

Lastly, the treatment options for cirrhotic ascites are still limited. Current approaches mainly involve diuretics, salt-restricted diets, and paracentesis to alleviate the symptoms of cirrhotic ascites, but these methods do not cure the underlying disease. Therefore, finding new treatment targets and approaches is an important direction for future research.

All in all, although progress has been made in understanding the mechanisms of cirrhotic ascites, there are still many unresolved issues. Future research should focus on the interactions between different mechanisms, as well as identifying new biomarkers and treatment methods to improve the prevention and treatment of cirrhotic ascites.

## Author contributions

ZL: Writing – review and editing, Writing – original draft, Visualization, Project administration, Methodology, Investigation, Formal analysis, Data curation, Conceptualization. JZ: Writing – review and editing, Project administration, Funding acquisition, Conceptualization. HO: Writing – original draft.
